# Ethnomedical survey of plants used by the Orang Asli in Kampung Bawong, Perak, West Malaysia

**DOI:** 10.1186/1746-4269-6-5

**Published:** 2010-02-07

**Authors:** Anbu Jeba Sunilson John Samuel, Anandarajagopal Kalusalingam, Dinesh Kumar Chellappan, Rejitha Gopinath, Suraj Radhamani, Hj Azman Husain, Vignesh Muruganandham, Proom Promwichit

**Affiliations:** 1 School of Pharmacy, Masterskill University College of Health Sciences, Taman Kemacahaya 11, Jalan Kemacahaya, Cheras, Selangor, Malaysia; 2 Honorary Associate, School of Pharmacy, La Trobe University, Bendigo, Victoria 3552, Australia; 3 School of Biomedicine and Health, Masterskill University College of Health Sciences, Taman Kemacahaya 11, Jalan Kemacahaya, Cheras, Selangor, Malaysia

## Abstract

**Background:**

A qualitative ethnomedical survey was carried out among a local Orang Asli tribe to gather information on the use of medicinal plants in the region of Kampung Bawong, Perak of West Malaysia in order to evaluate the potential medicinal uses of local plants used in curing different diseases and illnesses.

**Methods:**

Sixteen informants ranging in age from 35 to 65 years were interviewed. A total of 62 species of plants used by Orang Asli are described in this study based on field surveys and direct face to face communication. These plants belonged to 36 families and are used to treat a wide range of discomforts and diseases.

**Results:**

The results of this study showed that majority of the Orang Asli, of Kampung Bawong are still dependent on local plants as their primary source of medication. As the first ethnomedical study in this area, publishing this work is expected to open up more studies to identify and assess the pharmacological and toxicological action of the plants from this region.

**Conclusions:**

Preservation and recording of ethnobotanical and ethnomedical uses of traditional medicinal plants is an indispensable obligation for sustaining the medicinal and cultural resource of mankind. Extensive research on such traditional plants is of prime importance to scientifically validate their ethnomedical claims.

## Background

The study of tribal knowledge of plants is an imperative facet of ethnomedical research. People healed themselves with traditional herbal medicines and ancient remedies from time immemorial [1,2]. Human beings have found remedies within their habitat, and have adopted different strategies depending upon the climatic, phyto-geographic and faunal characteristics, as well as upon the peculiar culture and socio-structural typologies [3]. Most of such information is passed on to the following generations by traditional healers through oral communication and discipleship practice [4]. Moreover, the World Health Organization (WHO) has reported that about 80% of the world population relies on traditional medicine to cure ailments [5,6]. Plants play a major role in the treatment of diseases and still remain the foremost alternative for a large majority of people [7-9]. This knowledge, if wisely utilized, could draw out promising herbal leads [10].

**Figure 1:**
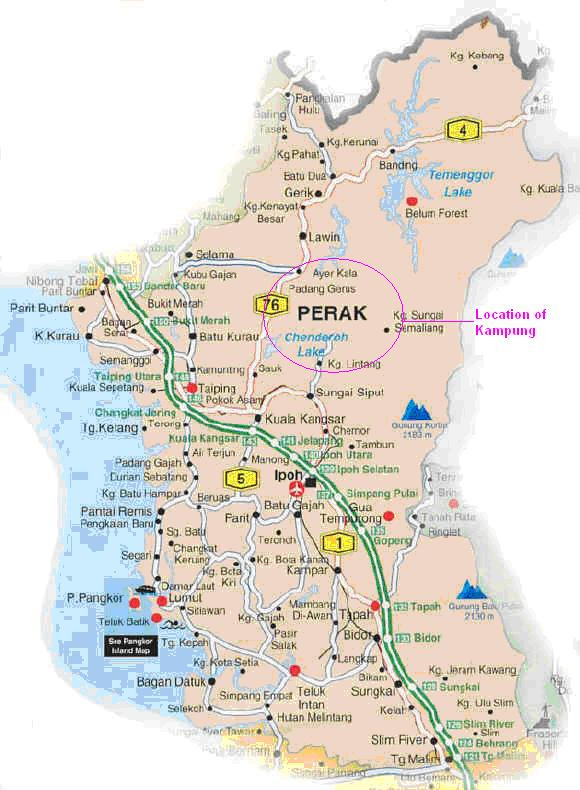
**Map of the Kampung Bawong region where the ethnomedical field survey was conducted**.

Perak, (Fig. 1) (5.02 N latitude and 101.08 E longitude), in Malaysia is one such area where traditional healing systems are still in practice among the local natives, especially the 'Orang Asli' tribes. Till date, no literature is available regarding the ethnomedical knowledge of this area, though there are ethnomedical reports on few other regions in Malaysia [11-13]. The 'Orang Asli', which means 'first people', are considered to be the original natives of peninsular Malaysia. There are about 150, 000 Orang Asli people of which 60% still live in the rain forests. There are 19 sub-groups among them, like Semai, Temiar, Lanoh and Jah Hut to name a few [14]. Many of the Orang Asli practitioners use local plant parts and plant juices to cure ailments and this practice is still in use [15]. Yet, little attention has been given to their traditional expertise to incorporate their knowledge in modern medicine. This study is an attempt to identify and document the use of traditional medicine among the local Orang Asli along the Kampung Bawong region in Perak.

## Methods

**Figure 2:**
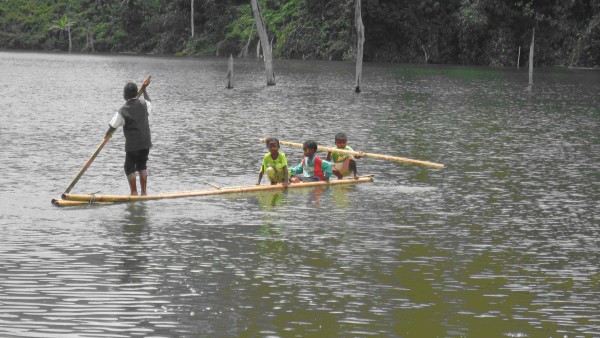
**An Orang asli crossing the river on their own wooden boat (perahuk) for fishing and hunting**.

**Figure 3:**
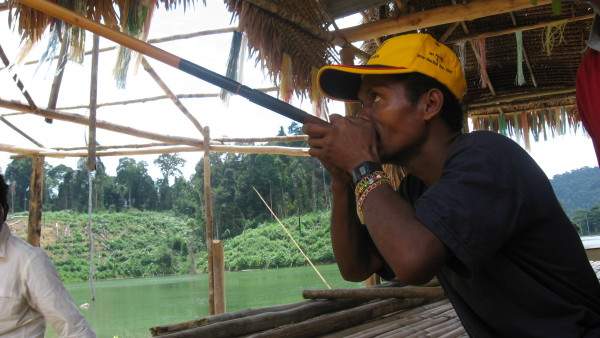
**An Orang asli using blow pipe made up of sewor bamboo for hunting**.

Regular field trips were made to the selected tribal localities in different seasons of the year 2008, conducted in rural area located in Kampung Bawong. The authors worked with a specific tribe of Orang asli called the 'semang' who fall under the group 'negrito' (Fig. 2, 3). Sixteen informants were involved in the interviews. All informants were in the age group of 35 to 65 years. All informants were male. 3 of them were practicing herbalists, and the rest 13 were individuals who gained knowledge on medicinal uses of plants from their parents and relatives who were historically using the plants with promising results. Interviews were conducted in a local dialect of Malay language. Interviewing individual informant was of fundamental importance to assure the reliability of the gathered information. Individual interviews were conducted with 7 informants (3 herbalists and 4 individual informants) and one group discussion involving the remaining 9 informants was also conducted. The interviews were built on trust with a common aspiration to improve the health situation in the country and to conserve and increase the knowledge on medicinal plants. The information was collected in the local dialect of Malay language. Special concern was taken in collecting information to steer clear of any unoriginal information by sources such as books and magazines were rejected. Some informants were repeatedly merited during field trips to confirm the information provided by them previously. Interpretation and translation of the information received into technical or medicinal terms was cautiously avoided during the interviews so as to obtain a genuine picture of customs and uses. All the plants were identified by Dr. Encik Sani, Botanist, Department of Botany, University Kebangsan Malaysia, Selangor, Malaysia. Voucher herbarium specimens were prepared and deposited in the herbarium of Department of Pharmacognosy, Masterskill University College of Health Sciences, Selangor, Malaysia.

## Results and Discussion

The present ethnomedical field survey indicated that there are 62 medicinal plant species belonging to a total of 36 families which are used in Kampung Bawong (Table 1). Most of these species grow in the wild naturally and their medicinal properties are crucial in traditional medicine of the Orang Asli. Majority of the species reported in this paper are widely known throughout peninsular Malaysia and are employed for a large number of medical conditions.

**Table 1:** Plants used by Orang Asli in Kampung Bawong, Perak of West Malaysia

Botanical Family	Botanical name	Local Malay Dialect	Part Used	Medicinal Uses
Acanthaceae	*Barleria lupulina *Lindl	Penawar Seribu Bisa	Leaves	Fresh leaves are used to remove warts
	
	*Barleria prionitis *Linn.	Hempedu landak	Leaves	Leaves are crushed and make into paste and applied over the inflamed area
	
	*Dipteracanthus repens *(L.) Hassk.	Deras malam	Leaves	Powder of dried leaves is mixed in warm water and drink to remove kidney stones
	
	*Eranthemum borneense *Hook f.	Sangsangkaruk	Leaves	Paste of leaves is applied to treat muscle cramps
	
	*Strobilanthes crispus *Blume.	Bayam karang	Leaves	Fresh leaves are masticated and swallowed as such to enhance the immune system

Annonaceae	*Annona muricata *Linn.	Durian Makkah	Leaves	Leaves are used to treat to kill all types of lice
	
			Fruits	Fruit juice is used to treat Stomach pain and hypertension
	
	*Uvaria sorsogonensis *C.Presl.	Segombong	Leaves	Decoction of the leaves is used to cure stomach ulcer

Araliaceae	*Arthrophyllum diversifolium *Blume.	Ondolus	Roots	Roots are boiled with water and drink to relieve body pain

Asteraceae	*Eupatorium odoratum *Linn.	Pokok kapalterbang	Leaves	Decoction of leaves is used as diuretic

Asclepiadaceae	*Hoya coronaria *Blume.	Takop	Leaves	Crushed leaves are applied to cure cuts and wounds

Bombacaceae	*Bombax ceiba *Linn.	Kapok	Leaves	Leaves are soaked into water and the decoction is taken for bath to treat body pain

Caesalpiniaceae	*Caesalpinia crista *Linn.	Gorek	Seeds	Seeds are crushed and mixed with sambal for appetite

Caprifoliaceae	*Sambucus javanica *Reinw. ex Blume	Kerak nasi	Leaves	Crushed the leaves with water and applied on inflamed parts to reduce pain and inflammation

Clusiaceae	*Garcinia mangostana *Linn.	Mangusta	Fruit	Fresh juice is used as nutrient drink
	
			Pericarp	Dried powder is used to heal the open wounds

Compositae	*Artemisia argyi *Levi. et Vant.	Ulam mak wan	Leaves	Fresh leaves are chewed in case of cough
	
	*Gynura procumbens *(Lour.) Merr.	Daun dewa	Leaves	Fresh leaves are used for to control blood glucose level

Connaraceae	*Agelaea macrophylla *(Zoll.) Leenh.	Akar pinang kutai	Leaves	The paste of leaves is used to treat acne
	
	*Cnestis platantha *Griff.	Binsangut	Leaves	Young leaves are warmed and applied to treat high fever

Euphorbiaceae	*Croton caudatus *Geisel	Tapasan komudi	Roots	Roots are boiled and the infusion is used as Nutrition.
	
	*Euphorbia tirucalli *Linn.	Mentulang	Latex	Latex is used to remove warts
	
	*Jatropha curcas *Linn.	Jarak Belanda	Leaves	Paste of young leaf is applied to treat cuts and wounds
	
			Roots	Roots are boiled and infusion is taken to treat diarrhea
	
	*Phyllanthus niruri *Linn.	Dukung Anak	Whole plant	Decoction of whole plant is used to treat jaundice

Fabaceae	*Parkia speciosa *Hassk.	Petai	Seeds	Fresh seeds are cooked and used to treat kidney disorders

Gnetaceae	*Gnetum leptostachyum *Blume.	Langod-langod	Whole plant	The plant was boiled in water and drink for relieve fever and flu

Lauraceae	*Cassytha filiformis *Linn.	Cemara Puteri	Whole Plant	Concoction used for the treatment of impotency

Leguminosae	*Archidendron ellipticum *Blume.	Bulinat	Leaves	Leaves are used to kill lice
	
	*Bauhinia semibifida *Roxb.	Daup-daup	Roots	Roots are boiled and the infusion is used to treat fatigue
	
	*Peltophorum *pterocarpum (DC) K. Heyne	Cugah	Barks	Powdered barks are applied on the affected area to treat psoriasis
	
	*Pongammia pinnata *Linn.	Kacang kayu laut	Leaves and Seeds	Fresh leaves and seeds are crushed and applied to repel insects
	
			Barks	Decoction of barks is used to kill intestinal worms

Loranthaceae	*Dendrophoetoe constricta *Dans.	Salidan	Leaves	Paste of leaves is applied to treat headache

Malvaceae	*Abutilon indicum *Linn.	Kembang Lohor	Leaves	Poultice in the treatment of fever
	
	*Hibiscus rosa sinensis *Linn.	Bunga Raya	Root barks	Root barks is soaked in water for overnight and taken in empty stomach to treat ulcer
	
	*Hibiscus tiliaceus *Linn.	Daun baru	Barks	Dried powder is used to cure all types of sexually transmitted diseases

Meliaceae	*Aglaia odorata *Lour.	Pacar cina	Flowers	An infusion is used to reduce fever
	*Trichilia trijuga *Roxb	Kayu kaling	Barks	Fresh barks are crushed and the juice is applied to cure cuts and wounds

Menispermaceae	*Tinospora crispa *Linn.	Pokok patawali	Stem	Decoction of the stem is used to treat diabetes

Myrsinaceae	*Ardisia colorata *Roxb.	Pacar inai	Leaves	Decoction of the leaves is used to cure viral infections such as herpes zoster, measles
	
	*Ardisia crenata *Sims.	Mata Ayam	Whole Plant	The crushed juice is used to treat earaches and fever

Myrtaceae	*Syzygium cerina *Hend.	Bagu	Roots	Roots are boiled with water and drink as an energizer
	
	*Syzygium samarangenese *Blume.	Red Jambu	Leaves	Leaves are used to treat skin infections

Oleaceae	*Jasminum sambac *(L.) Ait	Kampupot	Leaves	Young leaves are soaked in cold water and drink to treat gallstones
	
			Roots	Roots are boiled and the infusion is taken to treat diabetes mellitus

Oxalidaceae	*Averrhoa bilimbi *Linn.	Tulod-ulod	Leaves	A cocktail of the leaves along with the fruit is used to treat Syphilis

Orchidaceae	*Bulbophyllum mutabile *(Bl.) Lindl	Tatau	Leaves	Leaves are boiled and the decoction is used to treat fever

Poaeceae	*Imperata cylindrica *(L.) Beauv.	Lalang	Whole plant	Dried powdered plant is applied over the wounds to prevent microbial infections

Portulacaceae	*Talinum triangulare *(Jacq.) Willd.	Akar singsum	Flowers	Powder of dried flowers is mixed with tea and drink to treat asthma

Rubiaceae	*Morinda citrifolia *Linn.	Bingkudu	Fruits	Fruits juice is used to treat Jaundice
	
	*Oldenlandia diffusa *(Willd.) Roxb.	Siku-siku	Leaves	Juice of fresh leaves is used as Sedative

Ruscaceae	*Sansevieria trifasciatai *Prain.	Snake plant	Leaves	2 or 3 drops of fresh juice is instilled into ear to reduce pain and inflammation

Sapotaceae	*Planchonella obovata *(R. Br.) Pierre	Gombirat	Leaves	A paste of the leaves is applied on the forehead to relieve headache

Schizaeaceae	*Lygodium circinnatum *Burm.	Ribu-ribu	Leaves	Infusion of leaves is used to cure eye infection

Simaroubaceae	*Eurycoma longifolia *Jack.	Tongkat ali	Roots	Decoction of roots with tea is used as sexual stimulant

Solanaceae	*Solanum nigrum *Linn	Terong meranti	Fruits and Leaves	Fruits and leaves are chewed to treat upper respiratory tract infections

Umbeliferae	*Centella asiatica *Linn.	Pegaga	Leaves	Leaves are boiled and the infusion is used for mother who just give birth

Verbenaceae	*Lantana camara *Linn.	Bunga Tahi Ayam	Leaves	Leaves are boiled with water and spray to repel insects
	
	*Stachytarpheta jamaicensis *Linn. Vahl.	Bunga malam	Whole plant	The whole plant was crushed with water and applied on the injured ligament to relieve the pain and inflammation

Zingiberaceae	*Curcuma petiolata *Roxb.	Temu Puteri	Rhizomes	Juice is used to cure stomach ache
	
	*Languas conchigera *Burkill	Lengkuas Kecil	Rhizomes	Minced rhizomes are used for digestion
	
	*Kaempferia galanga *Linn.	Cekur	Rhizomes	Juice of the rhizomes is used for the treatment of stomach pains and coughs
	
	*Zingiber ottensii *Valeton	Kunyit Terus Hitam	Rhizomes	The juice of the rhizomes is used to cure all types of bacterial infections

The plants were often used by most of the informants more or less for the same purpose, and with only slight variations in recipes. The plants are usually collected from wild. All species were easily recognized by the informants with their respective local Malay dialect names. Some of the plants commonly used belong to the family Euphorbiaceae, Acanthaceae, Leguminosae, Zingiberaceae and Malvaceae. Most of these plants were used to relieve pain and to cure wound. Certain plants have specific use such as *Strobilanthes crispus *Blume., which is used to enhance the immune system and *Eurycoma longifolia *Jack., roots used as aphrodisiac. Results of this survey indicate that these plants were in use for a long time by the ethnic group.

## Conclusions

This current ethnomedical field survey carried out among the Orang Asli living in the Kampung Bawong region of Perak, Malaysia reveals that many medicinal plants are still broadly used by the population in the area where the study was conducted for treating various diseases and ailments. It is believed that there are more than 100 species of traditional herbal medicines found in this region. Since many plant species are indicated as potential resource for treating various diseases, this should encourage further research in ethnomedicine. The informants' consensus in the treatment of the main reported diseases is quite high, giving more validity to the plants as a traditional remedy.

The current data will expand the genetic resources obtainable in the area of research and signify a potential source of natural products for treating various diseases. The preservation of these plant species is the gateway toward developing efficacious remedies for treating diseases. Due to lack of knowledge and interest among the younger generations, some of the traditional medical information was buried together with the previous generations. This implies that the local government and village authorities need to act fast to conserve the ethnomedical knowledge of Orang Asli in the village Kampung Bawong, and the medicinal plants require preservation in addition to the ethnobotanical and ethnomedical knowledge recording. The preservation of these herbs along with the traditional knowledge of how to use them is an indispensable obligation for sustaining traditional medicine as a medicinal and cultural resource. Thus a future extensive research of these plants in this locality is recommended to identify and assess their ethnomedical claim.

## Competing interests

The authors declare that they have no competing interests.

## Authors' contributions

All the authors interviewed Orang asli people and identified all plant material described. JAJS developed the concept, designed and lead the project and also reviewed the manuscript. KA, GR, HAH, RS, MV, DKC and PP conducted the survey about the plants used by Orang Asli. KA, DKC and GR were also involved in the preparation of manuscript. HAH and PP were also involved in the verification of collected plants data for their vernacular name. SR, DKC and MV were also involved in reviewing the manuscript. All authors read and approved the final manuscript.
